# Integrated metabolomic and transcriptomic profiling reveals lipid dysregulation and potential biomarkers in interstitial lung disease

**DOI:** 10.3389/fimmu.2026.1840447

**Published:** 2026-06-16

**Authors:** Ping Li, Ying Yan, Xia Zhao, Hongmei Yang, Shurong Bai, Honghong Ma, Xu Chen

**Affiliations:** 1 Department of Pulmonary and Critical Care Medicine, People’s Hospital of Ningxia Hui Autonomous Region, Yinchuan, China; 2 Department of Rheumatology and Immunology, People’s Hospital of Ningxia Hui Autonomous Region, Yinchuan, China

**Keywords:** biomarkers, interstitial lung disease, lipid metabolism, metabolomics, transcriptomics

## Abstract

Interstitial lung disease (ILD) comprises diverse chronic inflammatory and fibrotic disorders with poorly understood mechanisms and limited diagnostic biomarkers. Growing evidence implicates lipid metabolic reprogramming in ILD pathogenesis, yet integrated metabolomic–transcriptomic analyses remain scarce. We performed combined metabolomic and transcriptomic analysis using publicly available datasets. Plasma metabolite profiles of ILD and lobar pneumonia (LOB) patients were analyzed by NMR-based metabolomics. Multivariate analyses (PCA, PLS-DA, OPLS-DA) identified discriminatory metabolites. KEGG enrichment revealed associated pathways. Transcriptomic data from lung tissue were analyzed for differentially expressed genes, identifying 345 differentially expressed genes (|log2FC| > 0.585, adjusted p < 0.05), integrated with metabolomic data to identify shared pathways. ROC curves evaluated diagnostic performance of key metabolites. To validate the bioinformatic findings, we established a bleomycin (BLM)-induced ILD mouse model with or without high-cholesterol diet (HCD) intervention. RT-qPCR, Western blotting, H&E staining, and Masson’s trichrome staining were performed to assess gene expression and histopathological changes in lung tissue. Metabolomic profiling showed clear separation between ILD and LOB samples, driven by alterations in triglyceride-rich lipoproteins, phospholipids, and cholesterol fractions. Seven metabolites were significantly increased in ILD (p < 0.05). Integrated multi-omics identified “lipid and atherosclerosis” as a key shared pathway, encompassing six differential genes (CD36, NFKBIA, PIK3R1, SELP, CCL2, VCAM1) and one differential metabolite (Cholesterol [HDL4]). ROC analysis showed a combined metabolite model achieved AUC of 0.810. Experimental validation confirmed SELP, CCL2, and VCAM1 were upregulated while NFKBIA was downregulated in BLM-treated mice. HCD further aggravated BLM-induced pulmonary fibrosis and markedly elevated the expression of inflammatory cytokines (TNF-α, IL-6, IL-1β) as well as key pathway proteins (SELP, CCL2, VCAM1). This integrated multi-omics analysis reveals a strong link between lipid dysregulation and ILD pathogenesis. Cholesterol fractions, triglycerides, and phospholipids may serve as potential non-invasive biomarkers for ILD, while the lipid and atherosclerosis pathway represents a promising target for therapeutic intervention. Animal experiments further validated that HCD exacerbates ILD via the lipid and atherosclerosis pathway, reinforcing the clinical relevance of cholesterol dysregulation in ILD progression. Our findings provide new insights into the metabolic mechanisms of ILD and establish a foundation for future diagnostic and therapeutic development.

## 1 Introduction

Interstitial lung diseases (ILDs) comprise heterogeneous group of diffuse parenchymal lung disorders characterized by varying degrees of interstitial inflammation and fibrosis, leading to impaired gas exchange, progressive dyspnea, reduced exercise tolerance, and diminished quality of life (1, 2). Disease outcomes differ markedly among ILD subtypes: while some cases resolve spontaneously or remain stable, progressive fibrosing forms frequently result in respiratory failure and death (3, 4). Despite advances in clinical care, the underlying mechanisms of ILDs remain incompletely understood, posing challenges for early diagnosis and effective treatment (5–8).

Metabolomics and transcriptomics have provided novel perspectives on ILD pathogenesis. Metabolomic profiling of idiopathic pulmonary fibrosis (IPF) patients has revealed alterations in multiple metabolic pathways, including glycolysis, mitochondrial β-oxidation, tricarboxylic acid cycle, bile acid, heme, and glutamate/aspartate metabolism, suggesting that energy-demanding processes contribute to lung remodeling (9). In parallel, transcriptomic studies have identified key genes and signaling pathways involved in pulmonary fibrosis (10). For example, ubiquitin-specific protease 13 (USP13) is markedly reduced in IPF lung tissue, promoting fibroblast activation through PTEN ubiquitination and degradation (11–13). Likewise, IL-13Rα1 has been shown to modulate lung injury and repair in bleomycin-induced models, underscoring the role of immune-regulatory genes in disease progression (14).

Integrating metabolomics and transcriptomics enables a more comprehensive analysis of ILD pathogenesis at both metabolite and gene-expression levels (10, 15, 16). Such multi-omics approaches can reveal the interplay between metabolic pathways and gene regulation, identify novel biomarkers, and provide mechanistic insights beyond single-omics studies (16). Recent research in toxin-induced lung fibrosis has demonstrated the utility of combining gas chromatography–mass spectrometry metabolomics with transcriptomics to pinpoint critical pathways, such as arachidonic acid metabolism (17), highlighting the value of cross-platform integration. However, despite increasing interest in omics-based investigations of ILD and pulmonary fibrosis, most previous studies have focused on single-omics analyses or have examined only one molecular layer independently, limiting the understanding of biological cross-talk between systemic metabolic remodeling and local pulmonary transcriptional dysregulation (18–21). Moreover, integrated analyses combining plasma metabolomics with lung tissue transcriptomics remain relatively scarce in ILD research. Importantly, the potential role of lipid and cholesterol dysregulation as a convergent multi-omics mechanism in ILD has not been systematically investigated.

In this study, we performed an integrated metabolomic and transcriptomic analysis comparing ILD and lobar pneumonia (LOB) patients. By applying principal component analysis (PCA), partial least squares discriminant analysis (PLS-DA), and orthogonal PLS-DA (OPLS-DA), we identified key differential metabolites and pathways, with a particular focus on lipid and cholesterol metabolism. We further linked these metabolic signatures to transcriptomic changes and evaluated their diagnostic potential using receiver operating characteristic (ROC) analysis. To provide experimental validation of the bioinformatic findings, we employed a bleomycin-induced mouse model of ILD with and without high-cholesterol diet (HCD) supplementation to investigate whether elevated cholesterol exacerbates pulmonary fibrosis and activates the lipid and atherosclerosis pathway *in vivo*. Our findings provide novel insights into lipid metabolic dysregulation in ILD and highlight potential biomarkers and pathways for early diagnosis and therapeutic intervention.

## 2 Methods

### 2.1 Data sources

Metabolomic data were obtained from the Metabolomics Workbench database (https://www.metabolomicsworkbench.org) using the ST003674 dataset, which included plasma samples from 24 patients with interstitial lung disease (ILD) and 21 patients with lobar pneumonia (LOB), without subtype specification. This dataset contained 24 metabolites and 114 lipoprotein parameters measured by NMR-based metabolomics. In parallel, transcriptomic data were retrieved from the Gene Expression Omnibus (GEO) database (accession number GSE5774, https://www.ncbi.nlm.nih.gov/geo/query/acc.cgi?acc=GSE5774). GSE5774 is a bulk transcriptomic microarray dataset generated using the GPL4255 platform (NIH-NIEHS/Agilent Human Familial IIP 43K array), which comprised 52 ILD lung tissue samples and 18 normal lung tissue samples; however, specific subclassifications (e.g., IPF vs. NSIP) are not provided in the dataset metadata.

### 2.2 Data preprocessing

Metabolomic data were preprocessed using R (v4.4.1). The limma and impute packages were employed for data curation and imputation. Specifically, metabolites with >50% missing values were removed, and the remaining missing values were imputed using the k-nearest neighbor (KNN) algorithm to improve data completeness. The data were then median-normalized to minimize technical variation.

### 2.3 Principal component analysis

Principal component analysis (PCA) was performed to evaluate overall distribution patterns and sample similarity. After log2 transformation where appropriate, data were transposed and analyzed using the prcomp function in R. The first two principal components (PC1 and PC2) were visualized with the ggplot2 and ggrepel packages, including 95% confidence ellipses for each group to illustrate clustering trends between ILD and LOB samples.

### 2.4 Partial least squares–discriminant analysis

To identify group-discriminating metabolic features, PLS-DA was conducted using the ropls package with two principal components. Model performance was evaluated using 7-fold cross-validation, and leave-one-out cross-validation was additionally assessed to examine model stability. The predictive ability and goodness-of-fit of the model were assessed using R²Y and Q²Y metrics. Model robustness was evaluated with 100 permutation tests. The final 2-component PLS-DA model achieved R²Y = 0.487 and Q²Y = 0.312, and permutation testing confirmed the statistical validity of the model (pR²Y = 0.04, pQ² = 0.02). Score plots and variable importance in projection (VIP) scores were generated to identify metabolites with the greatest contributions to group separation.

### 2.5 Orthogonal partial least squares–discriminant analysis

OPLS-DA, an extension of PLS-DA designed to remove orthogonal variation unrelated to class separation, was also performed using the ropls package. One predictive and one orthogonal component were set, and model validity was assessed with 1,000 permutation tests. Model performance was evaluated using R²X, R²Y, and Q²Y parameters. VIP plots were used to screen metabolites contributing most significantly to group discrimination.

### 2.6 Identification of differential metabolites

Differential metabolites between ILD and LOB groups were identified using the limma package. Metabolites with p < 0.05 and VIP > 1 were considered significant. Heatmaps, volcano plots, and violin plots were generated to visualize the expression patterns of significant metabolites across groups.

### 2.7 Receiver operating characteristic curve analysis

Diagnostic performance of individual and combined metabolites was assessed using the pROC and glmnet packages. The area under the curve (AUC) with 95% confidence intervals was calculated to evaluate the discriminatory ability of each metabolite or metabolite panel for distinguishing ILD from LOB samples.

### 2.8 KEGG pathway enrichment analysis of differential metabolites and genes

Differential metabolites were annotated using KEGG pathway information retrieved from MetaboAnalyst 6.0 (https://www.metaboanalyst.ca/). Pathway enrichment analysis was performed using the clusterProfiler package with a significance threshold of p < 0.05. For transcriptomic data (GSE5774), differential expression analysis was performed using the criteria |log2 fold change| > 0.585 and adjusted p < 0.05. Gene symbols were converted to Entrez IDs using the org.Hs.eg.db package. KEGG enrichment analysis was conducted with the enrichKEGG function in clusterProfiler, specifying Homo sapiens (“hsa”) as the reference organism. Integrated multi-omics pathway analysis was visualized with Venn diagrams generated using the ggvenn package to identify overlapping pathways between metabolomic and transcriptomic data. Bar plots and bubble plots of enriched pathways were generated with ggplot2, and key pathways were visualized using the pathview package to illustrate interactions between metabolites and genes.

The overall integration followed a pathway-centric strategy: (1) differential metabolites from plasma NMR metabolomics were subjected to KEGG pathway enrichment; (2) differentially expressed genes from lung tissue transcriptomics (GSE5774) were subjected to independent KEGG pathway enrichment; and (3) overlapping enriched pathways were identified via Venn diagram analysis, with shared pathways serving as biological convergence points for further investigation. This design is grounded in the principle that metabolites represent the downstream functional output of gene expression and enzymatic activity: plasma metabolomic changes reflect systemic metabolic reprogramming, while lung tissue transcriptomics captures the local gene regulatory landscape driving ILD pathology. Identifying pathways enriched in both layers thus enables the mechanistic “cross-talk” between systemic metabolic perturbations and local transcriptional dysregulation to be revealed.

### 2.9 Animal model and experimental design

To validate the bioinformatic findings *in vivo*, a bleomycin (BLM)-induced mouse model of ILD was established. Male C57BL/6 mice (8 weeks old) were randomly divided into three groups: Control, BLM, and BLM+HCD. Mice in the BLM and BLM+HCD groups received a single intratracheal instillation of BLM (3 mg/kg) to induce pulmonary fibrosis. The BLM+HCD group was simultaneously fed a high-cholesterol diet (HCD, containing 1.25% cholesterol) throughout the experiment to simulate cholesterol dysregulation observed in ILD patients. The Control group received an equivalent volume of normal saline and was maintained on standard chow. All animals were sacrificed at day 21 post-BLM administration. Lung tissues were collected for histological examination and molecular analyses. All animal procedures were approved by the Institutional Animal Care and Use Committee of People’s Hospital of Ningxia Hui Autonomous Region and conducted in accordance with relevant guidelines.

### 2.10 Hematoxylin and eosin and Masson’s trichrome staining

Lung tissues were fixed in 4% paraformaldehyde, embedded in paraffin, and sectioned at 4 μm. Hematoxylin and eosin (H&E) staining was performed to evaluate tissue morphology and inflammatory infiltration. Masson’s trichrome staining was conducted to assess collagen deposition and the degree of pulmonary fibrosis. Stained sections were examined under light microscopy and representative images were captured at low and high magnification.

### 2.11 RT-qPCR

Total RNA was extracted from mouse lung tissues using TRIzol reagent (Invitrogen). Complementary DNA (cDNA) was synthesized using a reverse transcription kit. Quantitative PCR was performed on a real-time PCR system using SYBR Green master mix. The relative mRNA expression levels of CD36 (Forward 5’-ATGACGTGGCAAAGAACAGCAGC-3’, Reverse 5’-GCAACAAACATCACCACTCCAATCC-3’), NFKBIA (Forward 5’-AGCAGACTCCACTCCACTTG-3’, Reverse 5’-GACATCAGCCCCACATTTCA-3’), PIK3R1 (Forward 5’-TATTGCGAGGGAAGCGAGAC-3’, Reverse 5’-ACTTCGCCGTCTACCACTAC3’), SELP (Forward 5’-ACTCGTCAAAAGTCGTCCGT-3’, Reverse 5’-ACCACTGTCACTTTGCCCTC-3’), CCL2 (Forward 5’-TTCCGATCCAGGTTTTTAAT-3’, Reverse 5’-GCAACAAACATCACCACTCCAATCC-3’), VCAM1 (Forward 5’-CCCTTGCTGAATGCAAGGA-3’, Reverse 5’-TGGGACCATTCCAGTCACTTC-3’), TNF-α (Forward 5’-TCTTCTCATTCCTGCTTGTGG-3’, Reverse 5’-ATGAGAGGGAGGCCATTTG-3’), IL-6 (Forward 5’-CAAAGCCAGAGTCCTTCAGAG-3’, Reverse 5’-AGCATTGGAAATTGGGGTAG-3’), and IL-1β (Forward 5’-CAGCCTTATTTCGGGAGTCTATTC-3’, Reverse 5’-TATCCCTTTGTTAACCCATCTGTA-3’) were calculated using the 2−ΔΔCt method with GAPDH (Forward 5’-CGACTTCAACAGCAACTCCCACTCTTCC-3’, Reverse 5’-TGGGTGGTCCAGGGTTTCTTACTCCTT-3’) as the internal reference gene. All experiments were performed in triplicate.

### 2.12 Western blotting

Total protein was extracted from lung tissues using RIPA lysis buffer supplemented with protease inhibitors. Protein concentrations were determined by BCA assay. Equal amounts of protein were separated by SDS-PAGE and transferred onto PVDF membranes. Membranes were blocked with 5% non-fat milk, then incubated overnight at 4 °C with primary antibodies against CD36 (1:500, DF13262, Affinity), NFKBIA (1:500, BM3932, BOSTER), PIK3R1 (1:500, bsm-52216R, bioss), SELP (1:500, DF13294, Affinity), CCL2 (1:500, 26161-1-AP, proteintech) and VCAM1 (1:500, bsm-52248R, bioss). After three washes with PBS, membranes were incubated with secondary antibodies (1:2000, ab6721, Abcam, Cambridge, UK) for 90 minutes at room temperature. Protein bands were visualized using an enhanced chemiluminescence (ECL) substrate kit (Beijing Labgic Biotechnology CO., LTD., Beijing, China) according to the manufacturer’s protocol and analyzed using ImageJ software (version 1.8; National Institutes of Health). GAPDH (1:10000, AB0037, Abways) was used as loading controls.

### 2.13 Statistical analysis

All statistical analyses in this study were performed using R software (v4.4.1) and SPSS 26.0. SPSS 26.0 statistical software was used to analyze the experimental data. Measurement data were expressed as mean ± standard deviation ( $\overset{–}{\text{x}}$ ± SD), preceded by normality test (Shapiro-Wilk method) and variance chi-square test (Levene method).A p-value less than 0.05 was considered statistically significant. For continuous variables, either the Student’s t-test (for normally distributed data) or the Wilcoxon rank-sum test (for non-normally distributed data) was applied. Categorical variables were compared using the chi-squared test. For animal experiments involving three groups, one-way ANOVA followed by Tukey’s *post hoc* test was used for multiple comparisons.

## 3 Results

### 3.1 Principal component analysis

**Figure 1:**
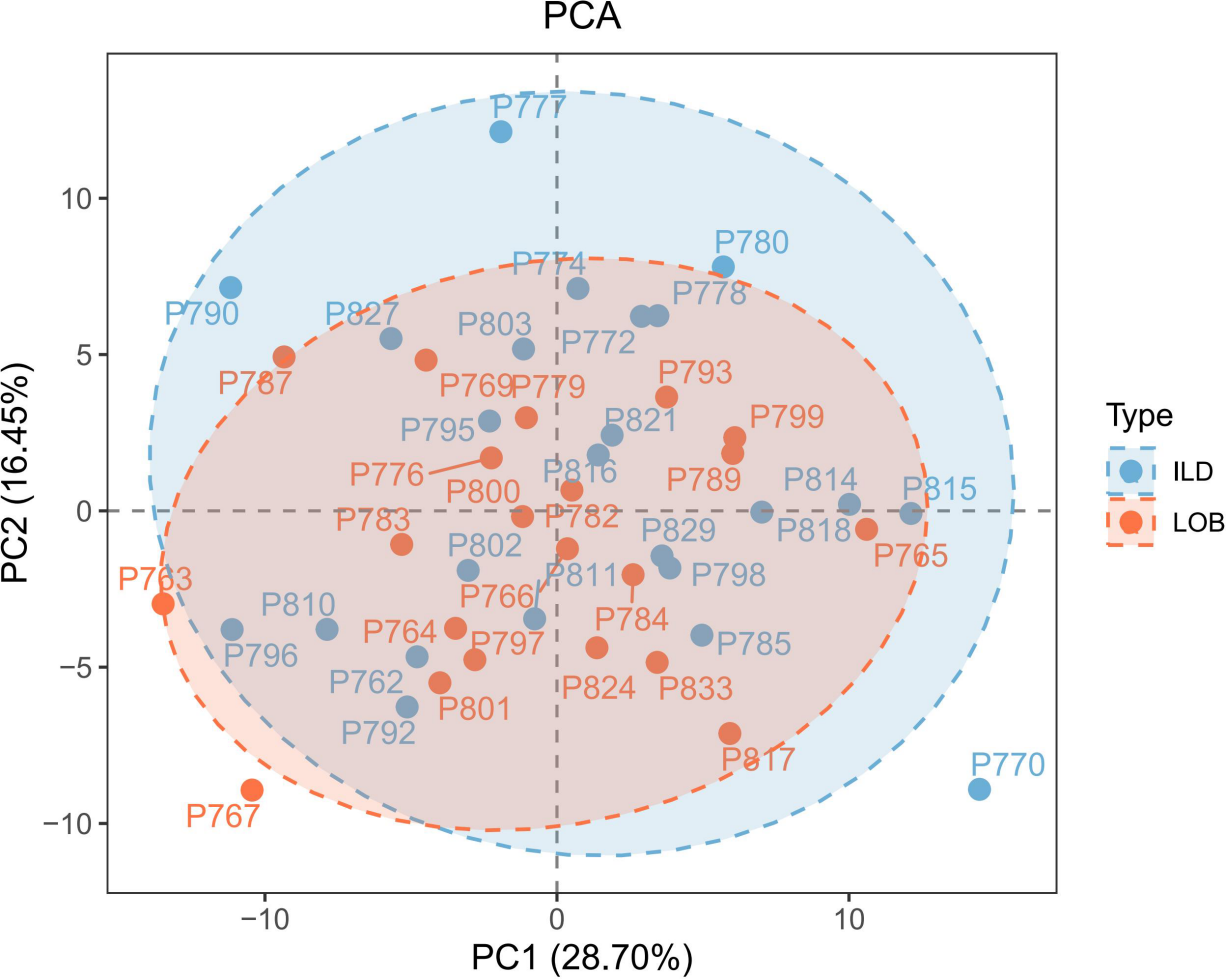
Principal component analysis (PCA) score plot of ILD and LOB plasma samples. Ellipses indicate the 95% confidence intervals for each group.

PCA was performed to assess the overall metabolic distribution between the ILD and LOB groups. As shown in **Figure 1**, PC1 and PC2 accounted for 28.70% and 16.45% of the total variance, respectively, cumulatively explaining 45.15% of the total variation. The score plot revealed a partial separation with notable overlap between ILD and LOB samples in the two-dimensional PC space. This pattern may reflect the biological heterogeneity inherent to ILD as well as shared metabolic features related to pulmonary inflammation common to both conditions. The 95% confidence ellipses illustrated the clustering tendency within each group.

### 3.2 Partial least squares–discriminant analysis

**Figure 2:**
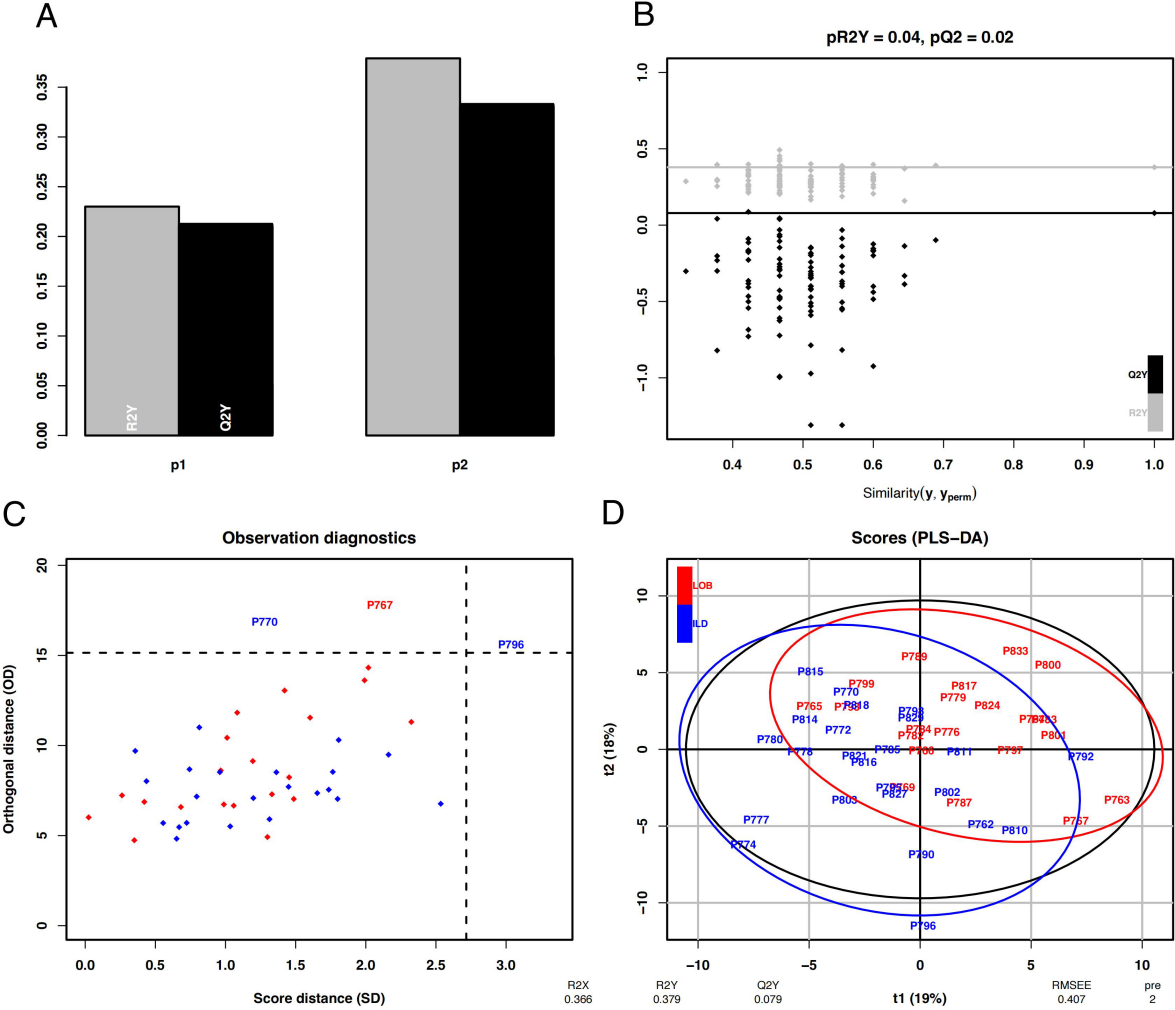
PLS-DA analysis. **(A)** Model explanatory and predictive ability plot (R²Y and Q²Y). **(B)** Permutation test of the PLS-DA model. **(C)** Diagnostic plot showing orthogonal and score distances to identify outliers. **(D)** PLS-DA score plot showing sample clustering by group.

A PLS-DA model with two principal components was constructed to explore discriminating features between groups. The model exhibited good explanatory and predictive abilities, as indicated by its R²Y and Q²Y values (**Figure 2A**). Permutation testing demonstrated statistical significance (pR2Y = 0.04, pQ2 = 0.02; **Figure 2B**). The diagnostic plot identified samples with high orthogonal distance (OD) or score distance (SD), including P796 (high OD) and P833, P789, P800 (extreme SD values; **Figure 2C**). The score plot showed a distinct clustering trend, with most LOB samples clustered on the right (P833, P789, P800), while most ILD samples clustered on the left (P777, P790, P796), indicating group separation facilitated by the supervised model (**Figure 2D**; **Supplementary Figure 1**).

### 3.3 Orthogonal partial least squares–discriminant analysis

**Figure 3:**
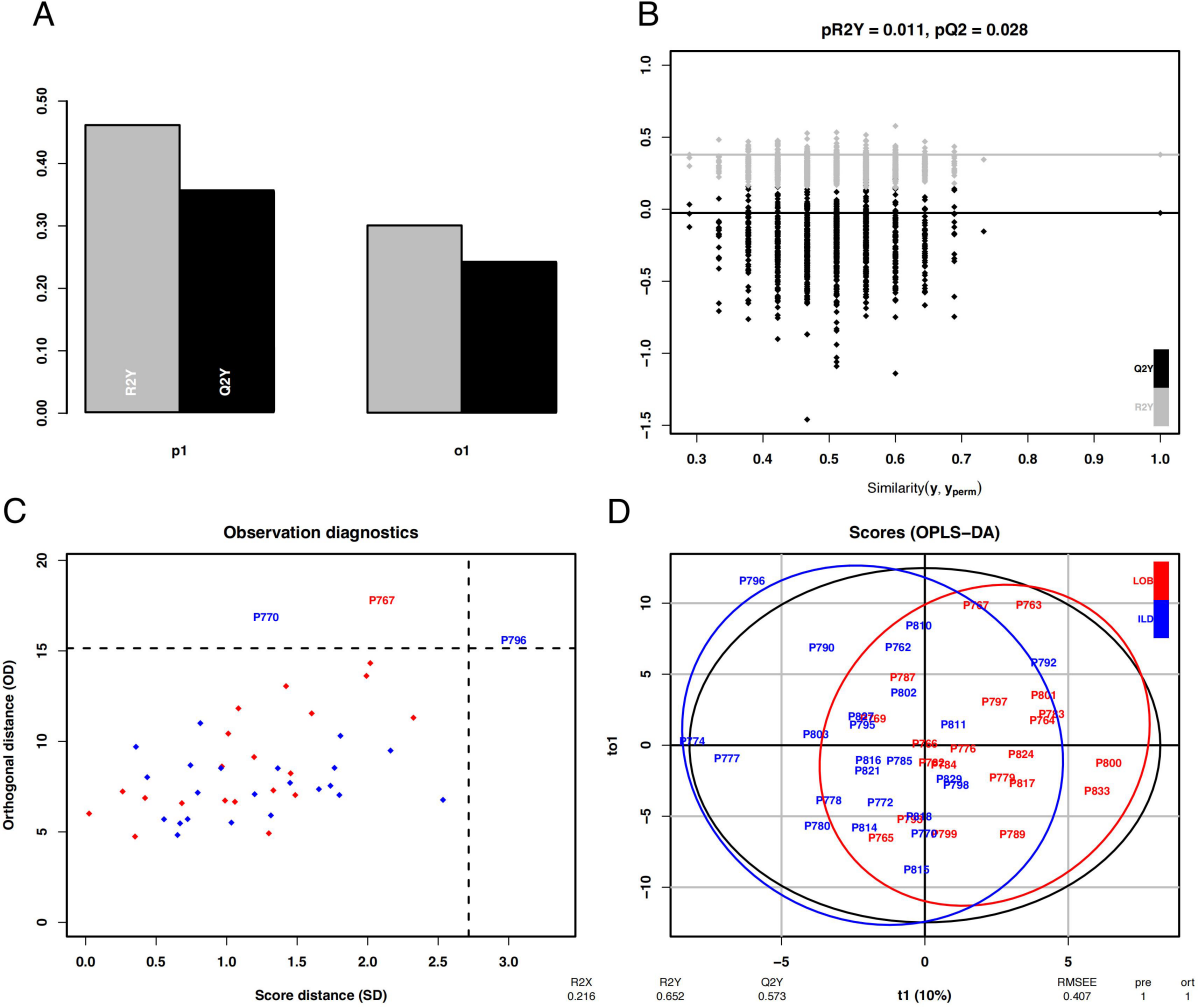
OPLS-DA analysis. **(A)** Model explanatory and predictive ability plot (R²Y and Q²Y). **(B)** Permutation test of the OPLS-DA model. **(C)** Diagnostic plot showing orthogonal and score distances. **(D)** OPLS-DA score plot showing improved separation between ILD and LOB samples.

OPLS-DA further improved group separation by removing orthogonal variation unrelated to classification. The model demonstrated strong explanatory (R²Y = 0.652) and predictive (Q²Y = 0.573) power (**Figure 3A**; **Supplementary Figure S2A**), with permutation testing confirming its robustness (pR2Y = 0.011, pQ2 = 0.028; **Figure 3B**). Diagnostic plots highlighted outliers with high OD values (e.g., P787 and P802) and key discriminating samples with high SD values (e.g., P796 and P767; **Figure 3C**). The score plot displayed clear group separation (**Figure 3D**). VIP analysis identified several metabolites with high contributions to group discrimination, including phospholipids (VIP = 2.27), triglycerides (VIP = 2.07), and free cholesterol (VIP = 2.02), suggesting their potential as biomarkers (**Supplementary Figure 2B**).

### 3.4 Differential metabolite analysis of ILD and LOB

**Figure 4:**
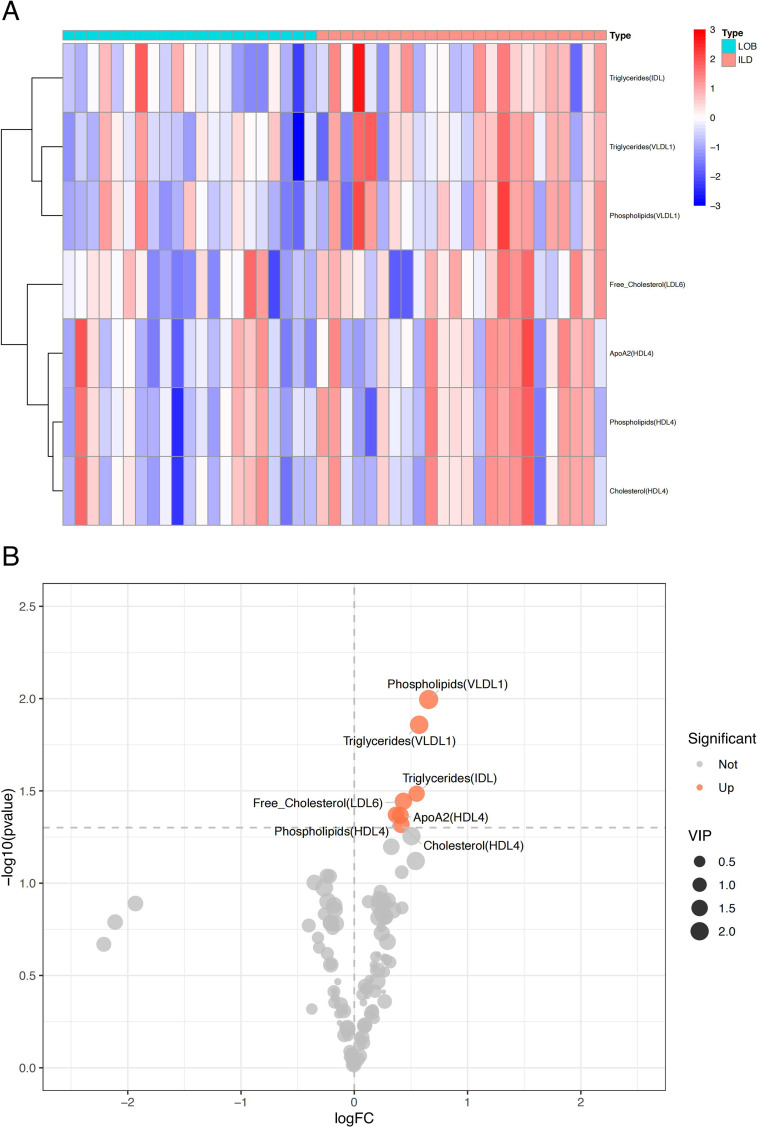
Differential metabolite profiles between ILD and LOB. **(A)** Heatmap of the seven differential metabolites between ILD and LOB samples. **(B)** Volcano plot showing the magnitude and statistical significance of differential metabolites.

A total of seven metabolites were significantly different between ILD and LOB samples, including Triglycerides (IDL), Triglycerides (VLDL1), Phospholipids (VLDL1), Free Cholesterol (LDL6), ApoA2 (HDL4), Phospholipids (HDL4), and Cholesterol (HDL4). All seven metabolites were upregulated in ILD samples. Heatmaps showed distinct expression patterns between groups (**Figure 4A**), and volcano plots illustrated the statistical significance and magnitude of differential expression (**Figure 4B**). Violin plots further depicted the distribution of each metabolite between the two groups (**Supplementary Figures 3A–G**).

### 3.5 Diagnostic performance of differential metabolites

ROC curve analysis showed that all seven differential metabolites achieved AUC values greater than 0.650, indicating modest discriminatory ability between ILD and LOB samples (**Supplementary Figures 4A–G**). A combined metabolite model yielded a markedly higher AUC of 0.810 (95% CI: 0.675–0.919; **Supplementary Figure 4H**), demonstrating improved and good discriminatory performance compared to individual metabolites (**Supplementary Figure 4I**).

### 3.6 KEGG pathway enrichment of differential metabolites and differentially expressed genes

**Figure 5:**
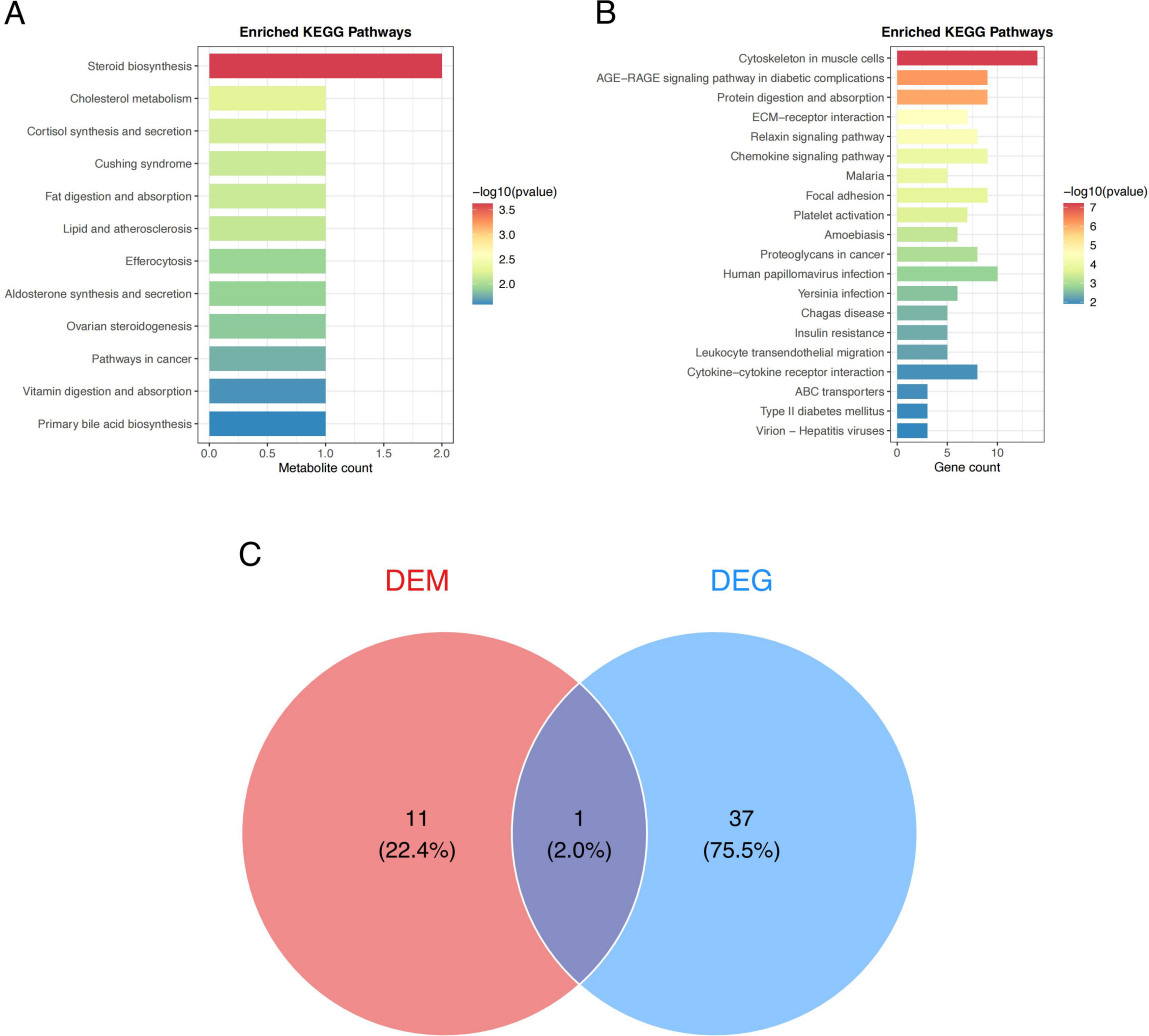
Pathway enrichment and cross-omics overlap analysis. **(A)** Bar plot of KEGG pathway enrichment for differential metabolites. **(B)** Bar plot of KEGG pathway enrichment for differentially expressed genes. **(C)** Venn diagram showing overlapping KEGG pathways between metabolomic and transcriptomic analyses.

KEGG enrichment analysis of the seven differential metabolites revealed 12 significantly enriched metabolic pathways (p < 0.05; **Table 1**). Cholesterol metabolism (hsa00260) showed the highest enrichment (fold=182.4), suggesting a critical role in disease development. Other enriched pathways included cortisol synthesis and secretion, Cushing syndrome, lipid digestion and absorption, and lipid/atherosclerosis pathways, indicating a strong association between lipid dysregulation and ILD pathogenesis. The bar plot highlighted steroid biosynthesis (hsa00071) as a key pathway, with two differential metabolites showing high significance (–log10(p) = 3.70; **Figure 5A**).

**Table 1:** Significantly enriched metabolic pathways matched by differential metabolites.

ID	Description	Rich factor	Fold enrichment	z-score	p-value	p.adjust	q value	gene ID	Count
hsa04979	Cholesterol metabolism	0.1000	182.4000	13.4518	0.0055	0.0179	0.0027	C00187	1
hsa04927	Cortisol synthesis and secretion	0.0833	152.0000	12.2696	0.0066	0.0179	0.0027	C00187	1
hsa04934	Cushing syndrome	0.0769	140.3077	11.7834	0.0071	0.0179	0.0027	C00187	1
hsa04975	Fat digestion and absorption	0.0769	140.3077	11.7834	0.0071	0.0179	0.0027	C00187	1
hsa05417	Lipid and atherosclerosis	0.0714	130.2857	11.3500	0.0077	0.0179	0.0027	C00187	1
hsa04148	Efferocytosis	0.0476	86.8571	9.2403	0.0115	0.0204	0.0031	C00187	1
hsa04925	Aldosterone synthesis and secretion	0.0455	82.9091	9.0241	0.0120	0.0204	0.0031	C00187	1
hsa04913	Ovarian steroidogenesis	0.0417	76.0000	8.6327	0.0131	0.0204	0.0031	C00187	1
hsa00100	Steroid biosynthesis	0.0351	64.0000	11.2265	0.0002	0.0034	0.0005	C01189/C00187	2
hsa05200	Pathways in cancer	0.0323	58.8387	7.5736	0.0169	0.0237	0.0036	C00187	1
hsa04977	Vitamin digestion and absorption	0.0238	43.4286	6.4766	0.0229	0.0291	0.0044	C00187	1
hsa00120	Primary bile acid biosynthesis	0.0213	38.8085	6.1094	0.0256	0.0299	0.0045	C00187	1

Transcriptomic analysis identified 345 differentially expressed genes between ILD and normal lung tissue (**Supplementary Table 1**). KEGG enrichment revealed 38 significantly enriched pathways (**Table 2**), with the top 20 pathways shown in **Figure 5B**. These included human disease–related metabolic pathways (e.g., parasitic diseases, endocrine and metabolic disorders) and signaling pathways (e.g., membrane transport, signaling molecules, and interactions). Cytoskeletal regulation in muscle cells (hsa04820) and human papillomavirus infection (hsa05165) showed the highest gene counts (n = 14 and 10, respectively). Linoleic acid metabolism (hsa00591) exhibited significant upregulation (p = 0.0356), suggesting involvement of lipid metabolic pathways in ILD progression.

**Table 2:** Significantly enriched metabolic pathways matched by differential gene.

Category	Subcategory	ID	Description	Gene ratio	Bg ratio	Rich factor	Fold enrichment	z-score	p-value	Gene ID	Count
Human Diseases	Infectious disease: parasitic	hsa05144	Malaria	5/94	50/9440	0.1000	10.0426	6.4292	0.0001	CD36/SELP/CCL2/VCAM1/COMP	5
Human Diseases	Endocrine and metabolic disease	hsa04933	AGE-RAGE signaling pathway in diabetic complications	9/94	101/9440	0.0891	8.9488	8.0543	0.0000	PRKCZ/F3/PIK3R1/FN1/CCL2/VCAM1/COL1A2/COL1A1/COL3A1	9
Organismal Systems	Digestive system	hsa04974	Protein digestion and absorption	9/94	105/9440	0.0857	8.6079	7.8617	0.0000	COL6A3/COL5A1/COL16A1/COL6A1/COL5A2/COL17A1/COL1A2/COL1A1/COL3A1	9
Environmental Information Processing	Signaling molecules and interaction	hsa04512	ECM-receptor interaction	7/94	89/9440	0.0787	7.8986	6.5576	0.0000	CD36/FN1/COL6A3/COL6A1/COL1A2/COMP/COL1A1	7
Environmental Information Processing	Membrane transport	hsa02010	ABC transporters	3/94	45/9440	0.0667	6.6950	3.8403	0.0100	ABCA3/ABCC8/ABCC3	3
Metabolism	Lipid metabolism	hsa00591	Linoleic acid metabolism	2/94	30/9440	0.0667	6.6950	3.1331	0.0356	PLA2G1B/CYP3A4	2
Human Diseases	Endocrine and metabolic disease	hsa04930	Type II diabetes mellitus	3/94	47/9440	0.0638	6.4101	3.7288	0.0113	ABCC8/PRKCZ/PIK3R1	3
NA	NA	hsa03272	Virion - Hepatitis viruses	3/94	48/9440	0.0625	6.2766	3.6754	0.0120	CLDN18/CLDN5/CLDN1	3
Organismal Systems	Endocrine system	hsa04926	Relaxin signaling pathway	8/94	130/9440	0.0615	6.1800	5.9641	0.0000	EDNRB/NFKBIA/ARRB1/PRKCZ/PIK3R1/COL1A2/COL1A1/COL3A1	8
NA	NA	hsa04820	Cytoskeleton in muscle cells	14/94	233/9440	0.0601	6.0342	7.8030	0.0000	ANKRD1/SPTBN1/TTN/FN1/COL6A3/COL5A1/COL6A1/COL5A2/ACTG2/FBLN2/COL1A2/COMP/COL1A1/COL3A1	14
Human Diseases	Infectious disease: parasitic	hsa05146	Amoebiasis	6/94	103/9440	0.0583	5.8500	4.9633	0.0005	GNA11/PIK3R1/FN1/COL1A2/COL1A1/COL3A1	6
Organismal Systems	Immune system	hsa04611	Platelet activation	7/94	126/9440	0.0556	5.5792	5.1894	0.0002	PRKCZ/RASGRP2/P2RY1/PIK3R1/COL1A2/COL1A1/COL3A1	7
Metabolism	Amino acid metabolism	hsa00350	Tyrosine metabolism	2/94	36/9440	0.0556	5.5792	2.7606	0.0497	PNMT/AOC3	2
Human Diseases	Infectious disease: parasitic	hsa05142	Chagas disease	5/94	103/9440	0.0485	4.8750	3.9656	0.0036	NFKBIA/GNA11/PIK3R1/BDKRB2/CCL2	5
Human Diseases	Cancer: specific types	hsa05217	Basal cell carcinoma	3/94	63/9440	0.0476	4.7822	3.0206	0.0247	HHIP/FZD5/FZD4	3
Organismal Systems	Immune system	hsa04062	Chemokine signaling pathway	9/94	193/9440	0.0466	4.6831	5.1844	0.0001	NFKBIA/PREX1/ARRB1/PRKCZ/RASGRP2/PIK3R1/CCL2/CCL13/CXCL14	9
Organismal Systems	Endocrine system	hsa04929	GnRH secretion	3/94	65/9440	0.0462	4.6350	2.9491	0.0268	ARRB1/GNA11/PIK3R1	3
Human Diseases	Endocrine and metabolic disease	hsa04931	Insulin resistance	5/94	109/9440	0.0459	4.6067	3.7981	0.0046	CD36/NFKBIA/PRKCZ/RPS6KA2/PIK3R1	5
Cellular Processes	Cellular community - eukaryotes	hsa04510	Focal adhesion	9/94	203/9440	0.0443	4.4524	4.9867	0.0002	EMP2/PIP5K1B/PIK3R1/FN1/COL6A3/COL6A1/COL1A2/COMP/COL1A1	9
Human Diseases	Infectious disease: bacterial	hsa05135	Yersinia infection	6/94	138/9440	0.0435	4.3663	3.9950	0.0025	NFKBIA/PIP5K1B/RPS6KA2/PIK3R1/FN1/CCL2	6
Organismal Systems	Immune system	hsa04670	Leukocyte transendothelial migration	5/94	116/9440	0.0431	4.3287	3.6176	0.0059	CLDN18/CLDN5/PIK3R1/CLDN1/VCAM1	5
Organismal Systems	Endocrine system	hsa04920	Adipocytokine signaling pathway	3/94	70/9440	0.0429	4.3040	2.7824	0.0324	CD36/NFKBIA/AGRP	3
Environmental Information Processing	Signaling molecules and interaction	hsa04061	Viral protein interaction with cytokine and cytokine receptor	4/94	100/9440	0.0400	4.0170	3.0417	0.0175	TNFSF14/CCL2/CCL13/CXCL14	4
Human Diseases	Cancer: overview	hsa05205	Proteoglycans in cancer	8/94	204/9440	0.0392	3.9383	4.2548	0.0009	FZD5/FZD4/TIMP3/PIK3R1/FN1/LUM/COL1A2/COL1A1	8
Environmental Information Processing	Signal transduction	hsa04064	NF-kappa B signaling pathway	4/94	105/9440	0.0381	3.8257	2.9200	0.0205	NFKBIA/TNFSF14/CCL13/VCAM1	4
Cellular Processes	Cellular community - eukaryotes	hsa04550	Signaling pathways regulating pluripotency of stem cells	5/94	144/9440	0.0347	3.4870	3.0159	0.0143	FZD5/ID1/FZD4/PIK3R1/TBX3	5
Environmental Information Processing	Signal transduction	hsa04668	TNF signaling pathway	4/94	119/9440	0.0336	3.3756	2.6154	0.0307	NFKBIA/PIK3R1/CCL2/VCAM1	4
Human Diseases	Infectious disease: viral	hsa05160	Hepatitis C	5/94	159/9440	0.0314	3.1580	2.7522	0.0210	NFKBIA/CLDN18/CLDN5/PIK3R1/CLDN1	5
Environmental Information Processing	Signaling molecules and interaction	hsa04514	Cell adhesion molecules	5/94	160/9440	0.0313	3.1383	2.7357	0.0216	CLDN18/CLDN5/SELP/CLDN1/VCAM1	5
Environmental Information Processing	Signal transduction	hsa04022	cGMP-PKG signaling pathway	5/94	166/9440	0.0301	3.0249	2.6396	0.0248	EDNRB/KCNMB4/GNA11/ADRB2/BDKRB2	5
Human Diseases	Infectious disease: viral	hsa05165	Human papillomavirus infection	10/94	333/9440	0.0300	3.0158	3.7557	0.0017	FZD5/FZD4/PRKCZ/PIK3R1/FN1/COL6A3/COL6A1/COL1A2/COMP/COL1A1	10
Organismal Systems	Circulatory system	hsa04270	Vascular smooth muscle contraction	4/94	134/9440	0.0299	2.9978	2.3358	0.0445	KCNMB4/PLA2G1B/GNA11/ACTG2	4
Cellular Processes	Cellular community - eukaryotes	hsa04530	Tight junction	5/94	170/9440	0.0294	2.9537	2.5778	0.0271	CLDN18/CLDN5/AMOTL2/PRKCZ/CLDN1	5
Human Diseases	Cardiovascular disease	hsa05415	Diabetic cardiomyopathy	6/94	205/9440	0.0293	2.9393	2.8152	0.0163	CD36/PRKCZ/PIK3R1/COL1A2/COL1A1/COL3A1	6
Environmental Information Processing	Signal transduction	hsa04310	Wnt signaling pathway	5/94	174/9440	0.0287	2.8858	2.5179	0.0296	FZD5/CTNNBIP1/FZD4/SERPINF1/MMP7	5
Human Diseases	Cardiovascular disease	hsa05417	Lipid and atherosclerosis	6/94	216/9440	0.0278	2.7896	2.6683	0.0206	CD36/NFKBIA/PIK3R1/SELP/CCL2/VCAM1	6
Environmental Information Processing	Signaling molecules and interaction	hsa04060	Cytokine-cytokine receptor interaction	8/94	298/9440	0.0268	2.6960	2.9835	0.0095	IL1RL1/ACVRL1/IL1RN/TNFSF14/CCL2/IL13RA2/CCL13/CXCL14	8
NA	NA	hsa04082	Neuroactive ligand signaling	5/94	199/9440	0.0251	2.5233	2.1780	0.0482	SLC6A4/AGRP/GNA11/ADRB2/P2RY1	5

### 3.7 Integrated multi-omics analysis

Venn diagram analysis revealed overlapping pathways between metabolomics and transcriptomics data, with lipid and atherosclerosis (hsa05417) emerging as a key shared pathway (**Figure 5C**). This pathway included six differential genes (CD36, NFKBIA, PIK3R1, SELP, CCL2, and VCAM1) and one differential metabolite (Cholesterol [HDL4]), suggesting synergistic regulation in ILD. Pathway visualization (**Supplementary Figure 5**) showed co-localization of three differential metabolites and six differential genes within the lipid and atherosclerosis pathway, underscoring the complementary insights from metabolomic and transcriptomic analyses.

### 3.8 Experimental validation of key pathway genes in the BLM mouse model

**Figure 6:**
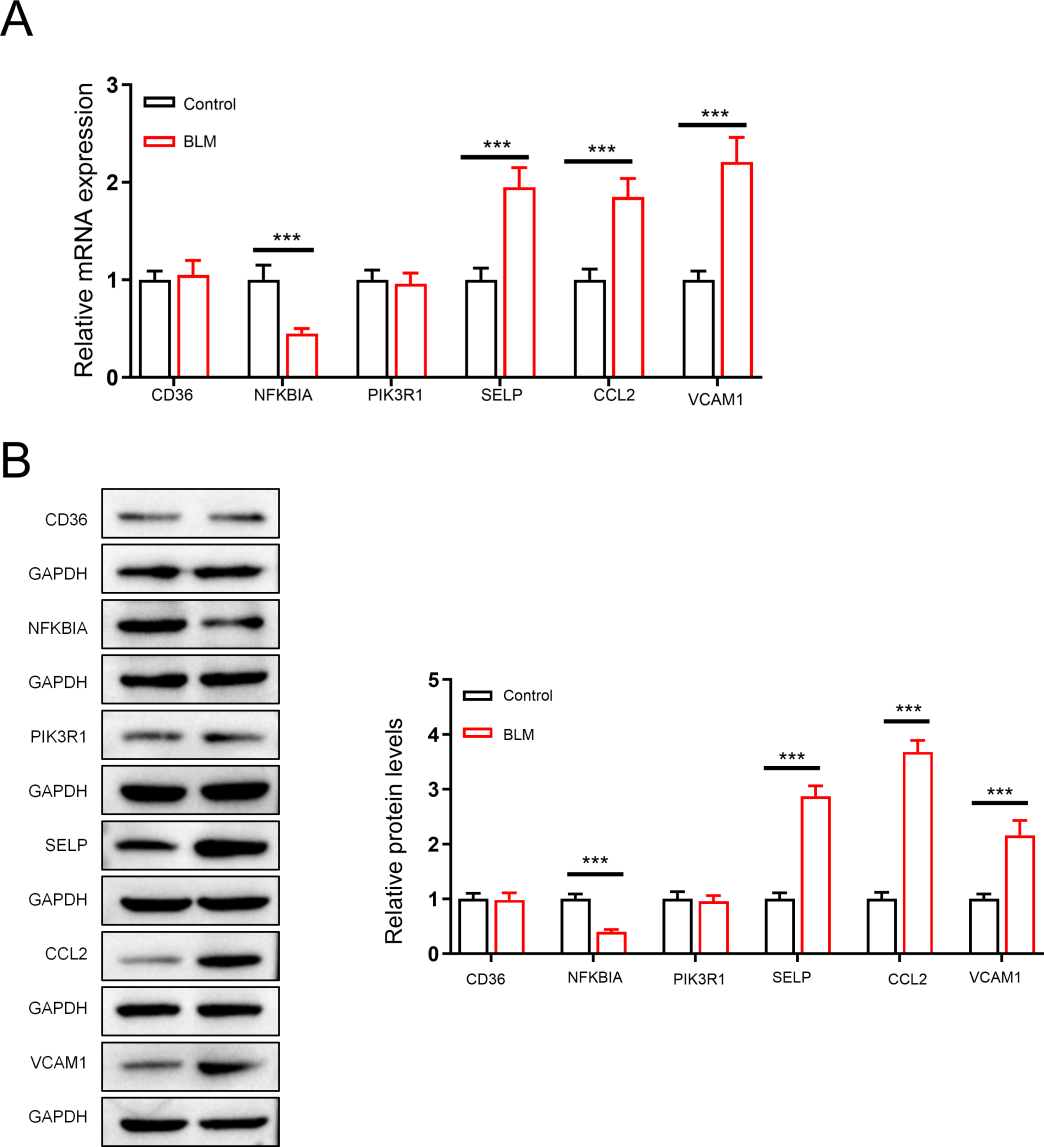
Validation of lipid and atherosclerosis pathway-related genes in bleomycin-induced ILD mice. **(A)** Relative mRNA expression levels of CD36, NFKBIA, PIK3R1, SELP, CCL2, and VCAM1. ***P < 0.001. **(B)** Western blotting analysis of CD36, NFKBIA, PIK3R1, SELP, CCL2, and VCAM1 protein levels. ***P < 0.001.

To validate the expression changes of the six candidate genes identified in the lipid and atherosclerosis pathway, we examined their mRNA and protein levels in lung tissues from BLM-treated mice. RT-qPCR analysis revealed that SELP, CCL2, and VCAM1 were significantly upregulated in the BLM group compared to the Control group, while NFKBIA was markedly downregulated (p < 0.001; **Figure 6A**). No significant differences were observed for CD36 or PIK3R1 at the mRNA level (p > 0.05). Western blotting confirmed these findings at the protein level: SELP, CCL2, and VCAM1 protein levels were significantly elevated in BLM-treated mice, whereas NFKBIA protein expression was significantly reduced compared to controls (p < 0.001; **Figure 6B**). CD36 and PIK3R1 protein levels remained comparable between groups, mirroring the mRNA results (p > 0.05; **Figure 6B**). These data validate the bioinformatic predictions and confirm that BLM-induced ILD is associated with dysregulation of key genes within the lipid and atherosclerosis pathway.

### 3.9 HCD exacerbates BLM-induced pulmonary fibrosis and inflammation

**Figure 7:**
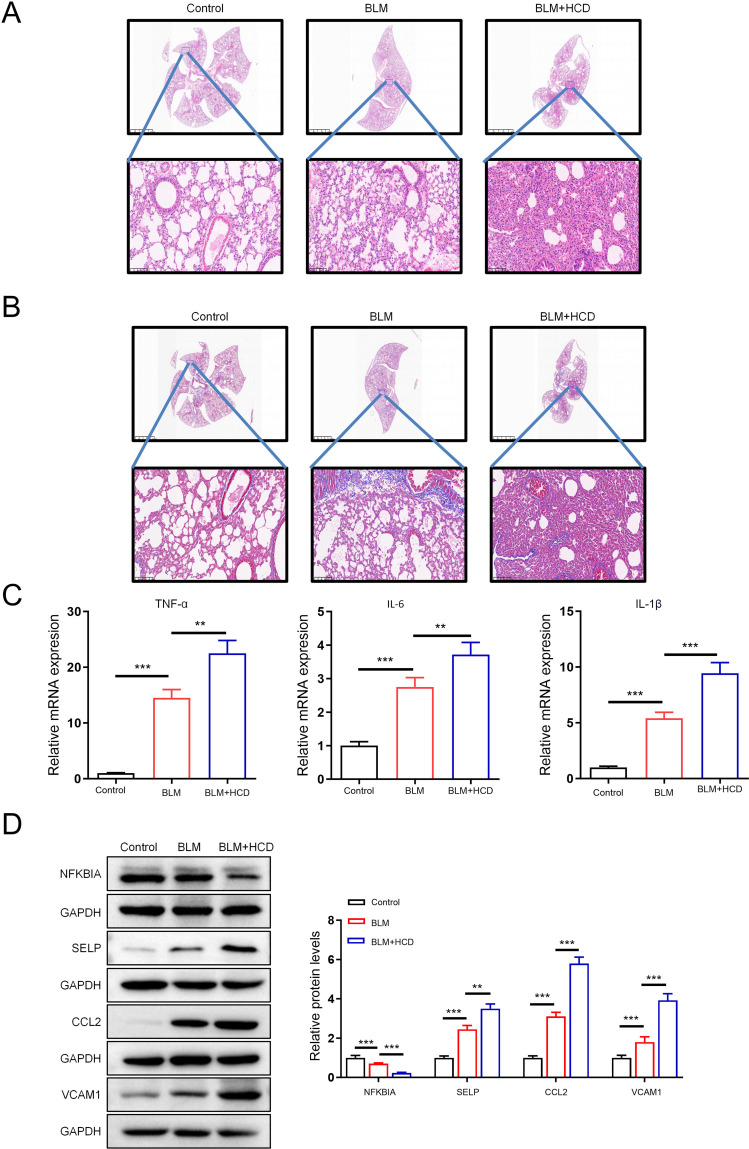
High-cholesterol diet aggravates lung injury, inflammation, and fibrosis in bleomycin-induced ILD mice. **(A)** Representative H&E staining and **(B)** Masson's trichrome staining of lung tissues from Control, BLM, and BLM+HCD groups. Scale bar = 0.5 mm (up graph) and 20 μm (down graph). **(C)** RT-qPCR of inflammatory cytokines (TNF-α, IL-6, and IL-1β) in different groups. **P < 0.01, ***P < 0.001. **(D)** Western blotting of NFKBIA, SELP, CCL2, and VCAM1 protein levels. **P < 0.01, ***P < 0.001.

To investigate whether elevated dietary cholesterol aggravates ILD, we subjected BLM-treated mice to high-cholesterol diet (HCD) and assessed pulmonary pathology and inflammation. H&E staining demonstrated that BLM treatment induced significant alveolar thickening, inflammatory cell infiltration, and disruption of normal lung architecture compared to controls. HCD further worsened these histopathological changes, with BLM+HCD mice displaying denser inflammatory infiltrates and more extensive alveolar damage (**Figure 7A**). Masson’s trichrome staining revealed substantially increased collagen deposition in BLM-treated lungs, which was further augmented in the BLM+HCD group, indicating that HCD promotes pulmonary fibrosis progression (**Figure 7B**). RT-qPCR showed that pro-inflammatory cytokines TNF-α, IL-6, and IL-1β were significantly elevated in the BLM group relative to controls, and their expression was further markedly increased in the BLM+HCD group (p < 0.01, p < 0.001; **Figure 7C**). Western blotting of NFKBIA, SELP, CCL2, and VCAM1 proteins corroborated the mRNA trends: SELP, CCL2, and VCAM1 were progressively upregulated from Control to BLM to BLM+HCD groups, while NFKBIA exhibited a stepwise decrease (**Figure 7D**). These results demonstrate that HCD not only aggravates pulmonary fibrosis but also amplifies the inflammatory response and dysregulates lipid and atherosclerosis pathway components, supporting a causal link between cholesterol metabolism and ILD pathogenesis.

## 4 Discussion

In this study, we conducted an integrated metabolomic and transcriptomic analysis to characterize the metabolic alterations and potential biomarkers associated with ILD. Using NMR-based metabolomics of plasma samples from ILD and LOB patients, combined with transcriptomic profiling of lung tissues, we identified distinct metabolic signatures and key pathways implicated in ILD pathogenesis. Our findings provide novel insights into lipid dysregulation and its interplay with gene expression, offering potential diagnostic and therapeutic targets.

Both unsupervised (PCA) and supervised (PLS-DA and OPLS-DA) multivariate analyses demonstrated clear metabolic distinctions between ILD and LOB samples. The clustering observed in PCA and the high R²Y and Q²Y values in PLS-DA and OPLS-DA models indicated robust group separation and predictive ability. Notably, we identified seven metabolites significantly upregulated in ILD, primarily involving triglyceride-rich lipoproteins, phospholipids, and cholesterol fractions. This lipid profile suggests alterations in lipoprotein remodeling, cholesterol efflux, and phospholipid turnover. Previous studies have shown that lipid mediators can influence pulmonary inflammation, surfactant composition, and fibroblast activation (22–26). Our KEGG enrichment analysis further confirmed that cholesterol metabolism and steroid iosynthesis are among the most significantly enriched pathways (27–30), supporting the hypothesis that dysregulated lipid metabolism may drive disease progression and immune responses in ILD.

Transcriptomic profiling identified 345 differentially expressed genes, with enrichment in 38 KEGG pathways. Notably, lipid-related pathways such as linoleic acid metabolism were significantly upregulated, further supporting the centrality of lipid dysregulation in ILD (10, 31). Pathways involved in cytoskeletal remodeling and immune regulation, including human papillomavirus infection and cytoskeletal regulation in muscle cells, may represent secondary effects of chronic inflammation and tissue remodeling. Together, these findings indicate that ILD pathogenesis involves coordinated changes in both lipid metabolism and immune-related signaling pathways.

Integration of metabolomic and transcriptomic data revealed lipid and atherosclerosis (hsa05417) as a key shared pathway, encompassing six differential genes (CD36, NFKBIA, PIK3R1, SELP, CCL2, and VCAM1) and one differential metabolite (Cholesterol [HDL4]). Many of these genes are involved in lipid transport, inflammation, and endothelial adhesion, suggesting a synergistic interaction between metabolic and transcriptional dysregulation. For example, CD36 is a scavenger receptor regulating fatty acid uptake and foam cell formation (32, 33), while VCAM1 and CCL2 are chemokines involved in leukocyte recruitment and vascular remodeling (34, 35). The co-localization of these features in the lipid and atherosclerosis pathway underscores its potential as a central mechanism linking metabolic reprogramming and chronic inflammation in ILD.

Our findings of dysregulated steroid biosynthesis and cortisol secretion pathways are consistent with previous metabolomic studies in idiopathic pulmonary fibrosis (IPF) and other interstitial lung diseases (36–38). Prior work has demonstrated that corticosteroid and lipid mediators modulate alveolar macrophage activation and fibroblast proliferation, thereby influencing tissue remodeling in the lung (39, 40). Notably, our multi-metabolite model (AUC = 0.810) outperformed single-metabolite markers, showed that combined lipidomic panels improve diagnostic accuracy in ILD. These convergent findings underscore the potential of lipid metabolism as both a biomarker source and a therapeutic entry point for ILD.

To experimentally validate the bioinformatic predictions, we established a BLM-induced mouse model of ILD. RT-qPCR and Western blotting analyses confirmed that SELP, CCL2, and VCAM1, three key components of the lipid and atherosclerosis pathway were significantly upregulated in BLM-treated lungs, while the anti-inflammatory regulator NFKBIA was downregulated. These changes align with the transcriptomic signature observed in human ILD tissue and support the pathological relevance of the identified pathway genes. Furthermore, introducing a high-cholesterol diet (HCD) in BLM-treated mice substantially worsened lung fibrosis and inflammation, as evidenced by more pronounced collagen deposition on Masson’s trichrome staining and elevated pro-inflammatory cytokines (TNF-α, IL-6, IL-1β). The further upregulation of SELP, CCL2, and VCAM1 proteins and additional suppression of NFKBIA under HCD conditions suggest that cholesterol overload amplifies inflammatory signaling and tissue remodeling in ILD. These findings are consistent with prior reports showing that high-fat or high-cholesterol diets can exacerbate lung injury and fibrosis (41–44), and provide direct mechanistic evidence that cholesterol metabolic dysregulation promotes ILD progression through the lipid and atherosclerosis pathway.

Our study has several strengths. First, we applied complementary multivariate models to ensure robust discrimination between groups. Second, we combined metabolomics and transcriptomics to uncover convergent pathways, thereby increasing biological interpretability. Third, we validated key pathway findings using an *in vivo* BLM mouse model with and without HCD intervention, bridging computational predictions with experimental evidence. Finally, we evaluated diagnostic potential using ROC analysis, demonstrating the clinical relevance of our findings. However, several limitations must be acknowledged. Our study was cross-sectional, limiting causal inference. The sample size was moderate, which may restrict the generalizability of our findings. Validation in independent cohorts and functional studies is warranted to confirm the biological roles of the identified metabolites and genes. Furthermore, the lack of detailed clinical metadata (e.g., age, sex, comedications) in the public datasets limits our ability to control for potential confounding factors.

## 5 Conclusion

Our integrated multi-omics approach reveals lipid metabolic dysregulation as a hallmark of ILD, identifies candidate biomarkers for early diagnosis, and highlights lipid and atherosclerosis as a key mechanistic pathway linking metabolism and gene expression. Experimental validation in a BLM-induced mouse model demonstrates that HCD further exacerbates pulmonary fibrosis, amplifies inflammation, and dysregulates core pathway genes (SELP, CCL2, VCAM1, and NFKBIA), providing direct *in vivo* evidence for the causal role of cholesterol dysregulation in ILD pathogenesis. These findings provide a basis for future mechanistic studies and open new avenues for biomarker development and targeted therapy in ILD.

## Data Availability

The datasets presented in this study can be found in online repositories. The names of the repository/repositories and accession number(s) can be found in the article/**Supplementary Material**.

## Supplementary material

Supplementary Figure 1 Enlarged PLS-DA score plot of ILD and LOB samples for clearer visualization of sample separation.

Supplementary Figure 2 OPLS-DA score plot and VIP score plot. (A) OPLS-DA score plot highlighting group separation. (B) VIP score distribution plot identifying the most discriminative metabolites.

Supplementary Figure 3 Distribution of Seven Key Differential Metabolites. (A–G) Violin plots showing the distribution of seven differential metabolites in ILD and LOB samples.

Supplementary Figure 4 Diagnostic Performance of Differential Metabolites and Combined Model. (A–G) ROC curves for individual differential metabolites. (H) ROC curve of the combined metabolite model. (I) Comparison of ROC curves between combined and individual metabolites.

Supplementary Figure 5 Visualization of the lipid and atherosclerosis pathway (hsa05417) showing co-localization of differential metabolites and genes.

Supplementary Table 1 The 345 differentially expressed genes between ILD and normal lung tissue.
