# Supplementary figures and images for: Integrated metabolomic and transcriptomic profiling reveals lipid dysregulation and potential biomarkers in interstitial lung disease
Source: Front Immunol. 2026 Jun 16;17:1840447. doi: 10.3389/fimmu.2026.1840447 (PMC13314440; doi:10.3389/fimmu.2026.1840447)

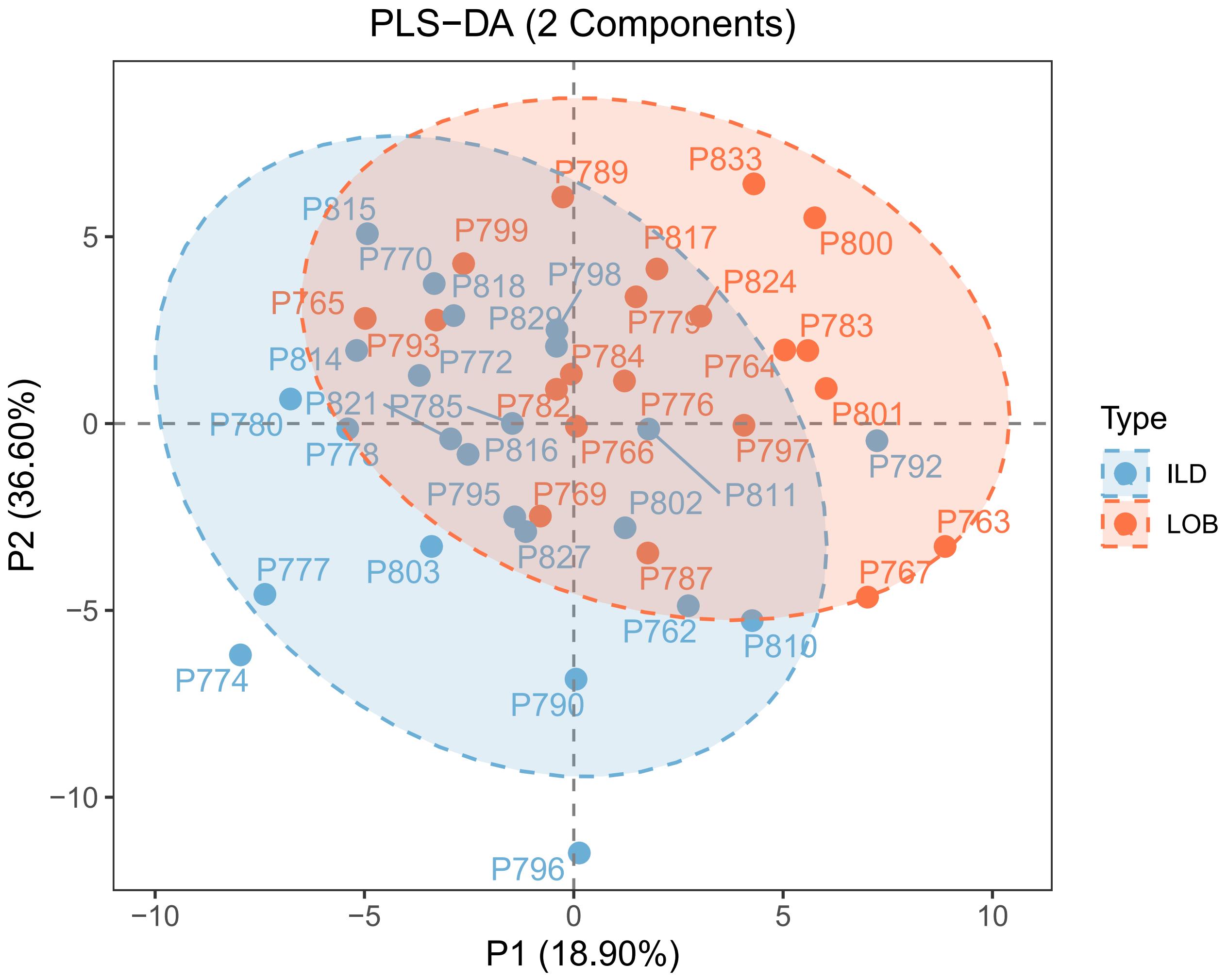

Supplement: Supplementary Figure 1 — Enlarged PLS-DA score plot of ILD and LOB samples for clearer visualization of sample separation. [file Image1.jpeg]

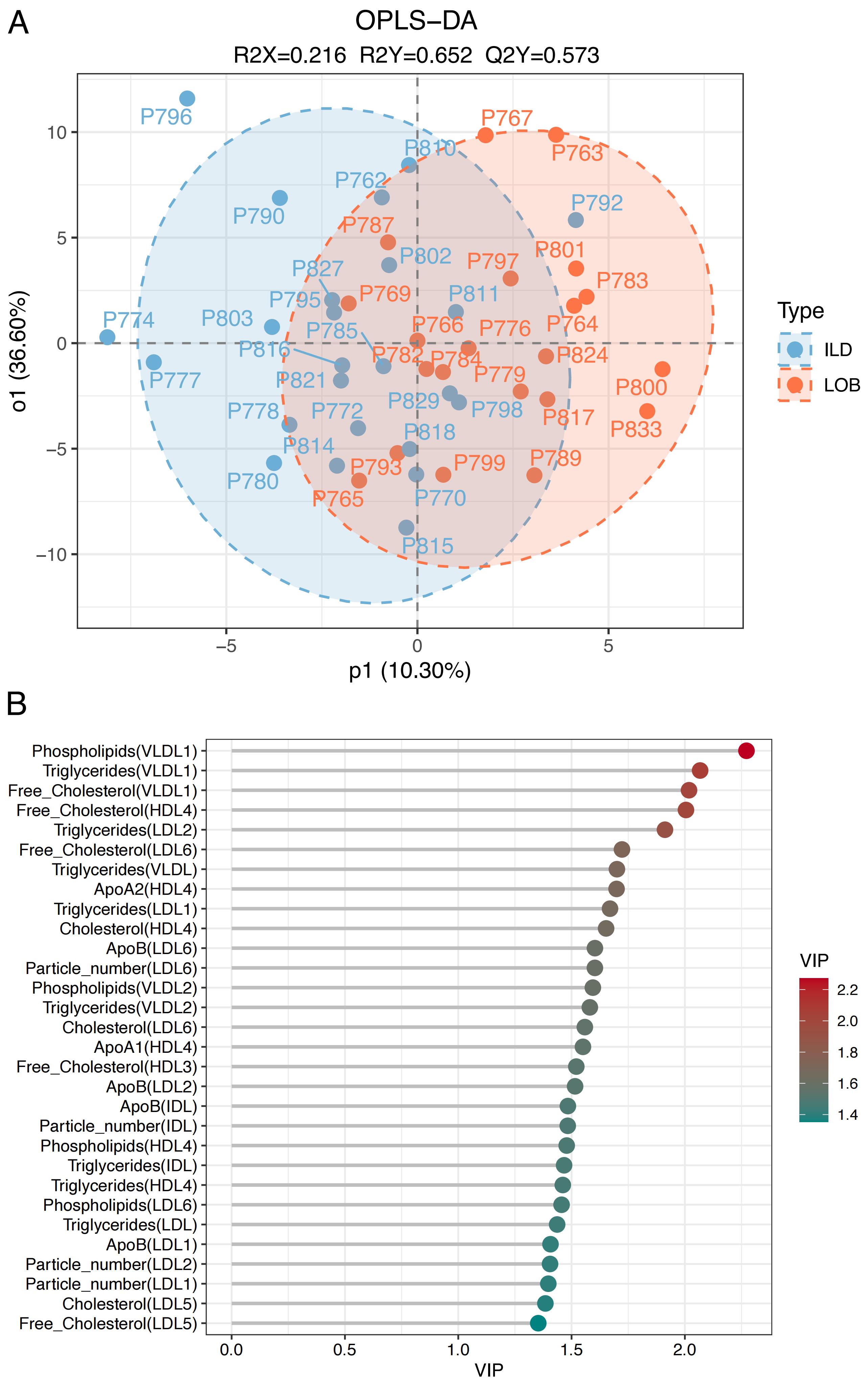

Supplement: Supplementary Figure 2 — OPLS-DA score plot and VIP score plot. (A) OPLS-DA score plot highlighting group separation. (B) VIP score distribution plot identifying the most discriminative metabolites. [file Image2.jpeg]

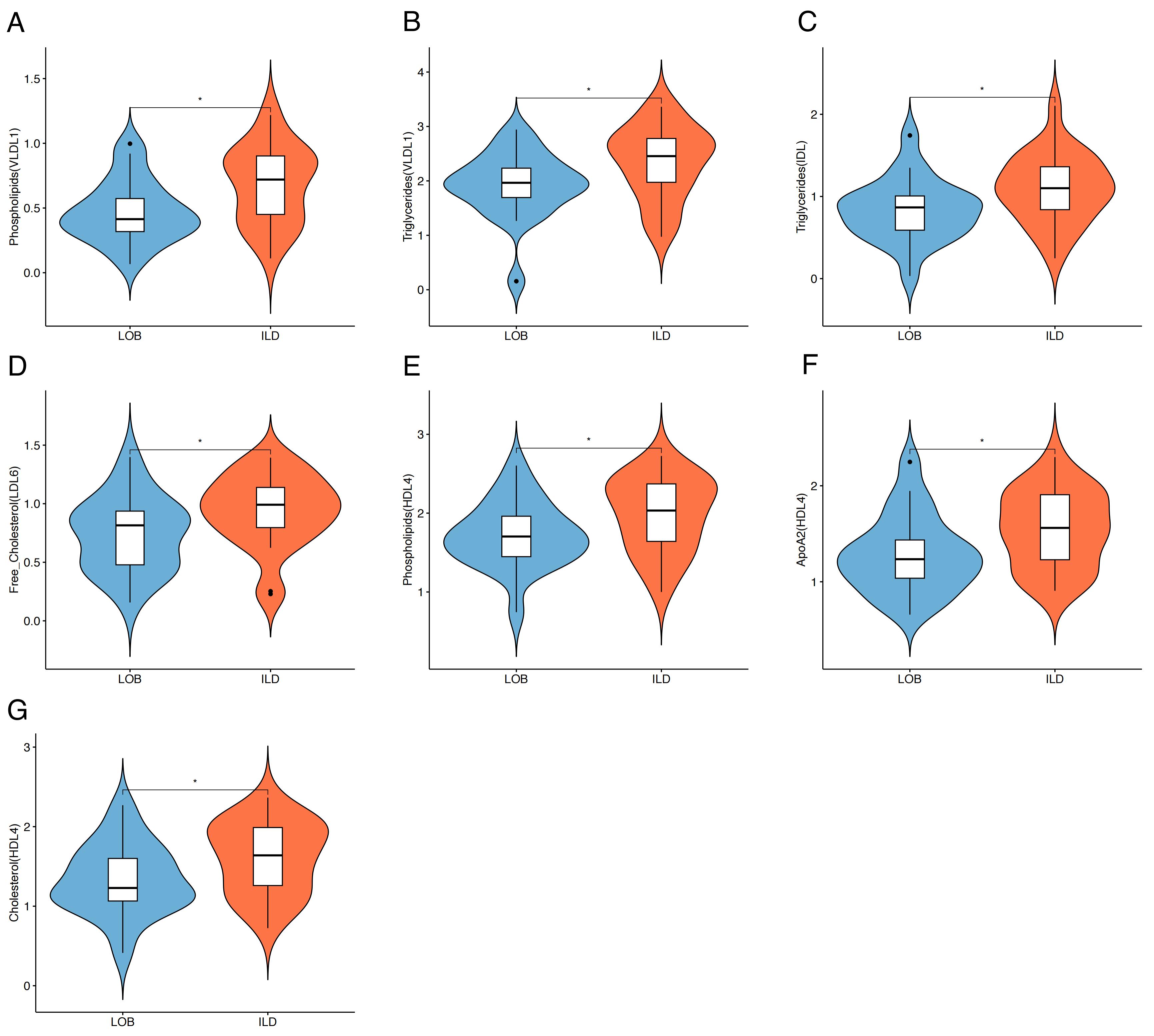

Supplement: Supplementary Figure 3 — Distribution of Seven Key Differential Metabolites. (A–G) Violin plots showing the distribution of seven differential metabolites in ILD and LOB samples. [file Image3.jpeg]

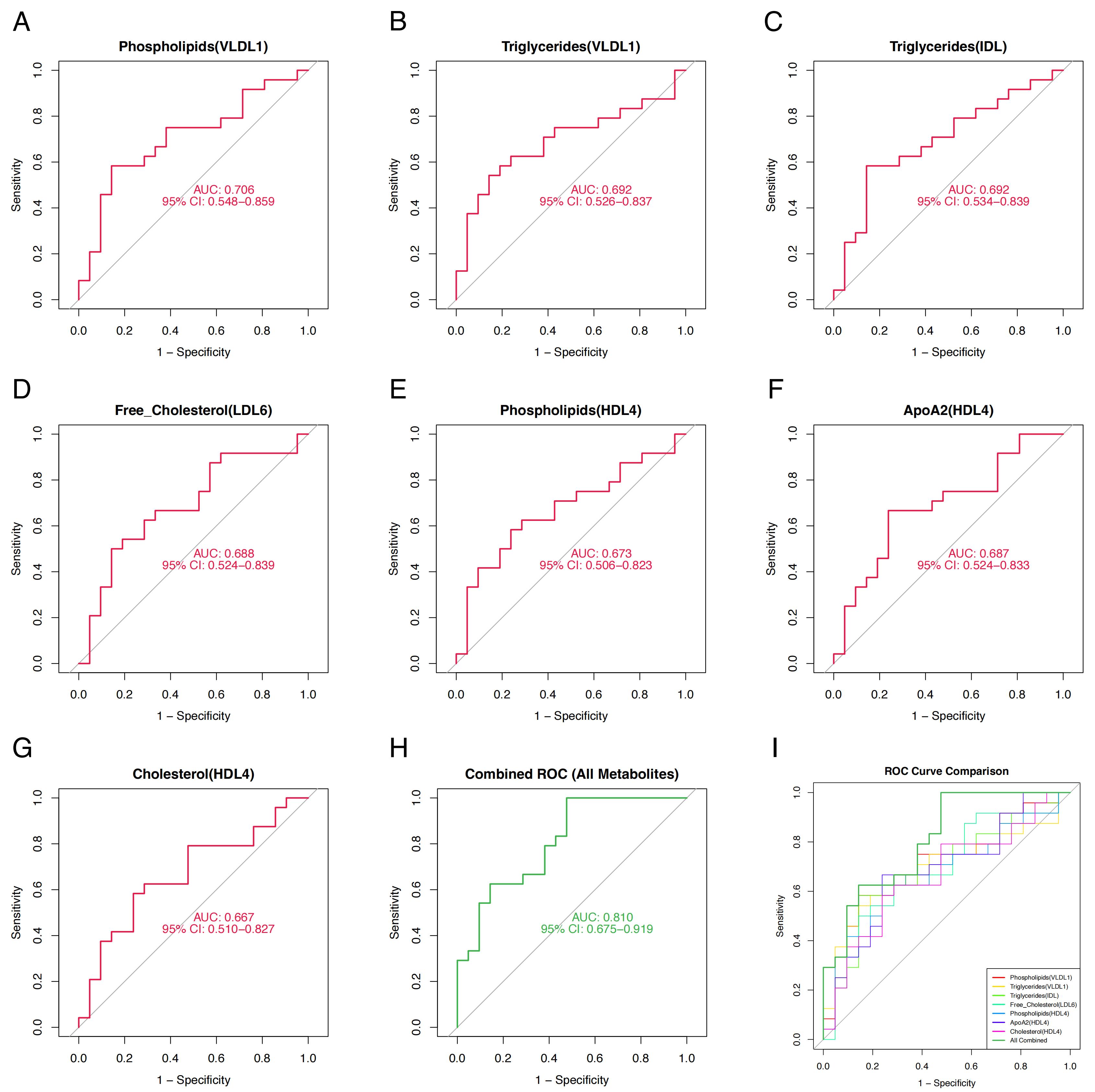

Supplement: Supplementary Figure 4 — Diagnostic Performance of Differential Metabolites and Combined Model. (A–G) ROC curves for individual differential metabolites. (H) ROC curve of the combined metabolite model. (I) Comparison of ROC curves between combined and individual metabolites. [file Image4.jpeg]

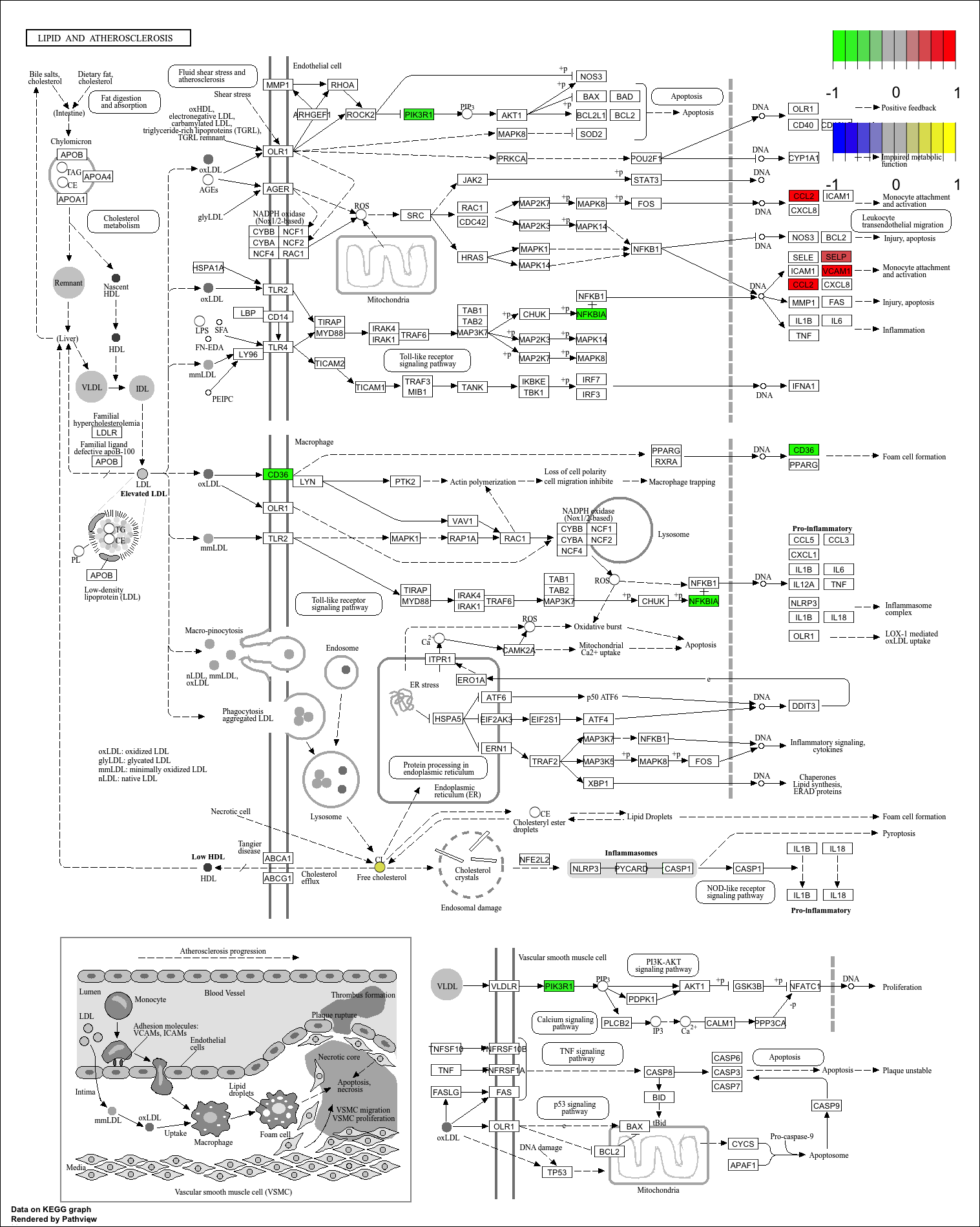

Supplement: Supplementary Figure 5 — Visualization of the lipid and atherosclerosis pathway (hsa05417) showing co-localization of differential metabolites and genes. [file Image5.jpeg]
